# Viral genomic, metagenomic and human transcriptomic characterization and prediction of the clinical forms of COVID-19

**DOI:** 10.1371/journal.ppat.1009416

**Published:** 2021-03-29

**Authors:** Christophe Rodriguez, Nicolas de Prost, Slim Fourati, Claudie Lamoureux, Guillaume Gricourt, Melissa N’debi, Florence Canoui-Poitrine, Isaac Désveaux, Oriane Picard, Vanessa Demontant, Elisabeth Trawinski, Raphaël Lepeule, Laure Surgers, William Vindrios, Jean-Daniel Lelièvre, Nicolas Mongardon, Olivier Langeron, José L. Cohen, Armand Mekontso-Dessap, Paul-Louis Woerther, Jean-Michel Pawlotsky

**Affiliations:** 1 Department of Microbiology, Hôpitaux Universitaires Henri Mondor, Assistance Publique-Hôpitaux de Paris (AP-HP), Créteil, France; 2 Team “Viruses, Hepatology, Cancer”, Institut Mondor de Recherche Biomédicale (IMRB), INSERM U955, Université Paris-Est, Créteil, France; 3 Médecine Intensive Réanimation, Hôpitaux Universitaires Henri Mondor, Assistance Publique-Hôpitaux de Paris (AP-HP), Créteil, France; 4 CARMAS Clinical Research Group, Université Paris-Est, Créteil, France; 5 Université Paris-Est-Créteil, INSERM, IMRB, Créteil, France; 6 Public Health and Clinical Research Unit, Hôpitaux Universitaires Henri Mondor, Assistance Publique-Hôpitaux de Paris (AP-HP), Créteil, France; 7 Department of Clinical Immunology and Infectious Diseases, Hôpitaux Universitaires Henri Mondor, Assistance Publique-Hôpitaux de Paris (AP-HP), Créteil, France; 8 Department of Anesthesiology and Surgical Intensive Care, Hôpitaux Universitaires Henri Mondor, Assistance Publique-Hôpitaux de Paris (AP-HP), Créteil, France; 9 Center for Clinical Investigation and Biotherapies, Hôpitaux Universitaires Henri Mondor, Assistance Publique-Hôpitaux de Paris (AP-HP), Créteil, France; 10 Team “Immunoregulation and biotherapies”, Institut Mondor de Recherche Biomédicale, INSERM U955, Université Paris-Est, Créteil, France; 11 Team DYNAMIC, Université Paris-Est, Créteil, France; Icahn School of Medicine at Mount Sinai, UNITED STATES

## Abstract

COVID-19 is characterized by respiratory symptoms of various severities, ranging from mild upper respiratory signs to acute respiratory failure/acute respiratory distress syndrome associated with a high mortality rate. However, the pathophysiology of the disease is largely unknown. Shotgun metagenomics from nasopharyngeal swabs were used to characterize the genomic, metagenomic and transcriptomic features of patients from the first pandemic wave with various forms of COVID-19, including outpatients, patients hospitalized not requiring intensive care, and patients in the intensive care unit, to identify viral and/or host factors associated with the most severe forms of the disease. Neither the genetic characteristics of SARS-CoV-2, nor the detection of bacteria, viruses, fungi or parasites were associated with the severity of pulmonary disease. Severe pneumonia was associated with overexpression of cytokine transcripts activating the CXCR2 pathway, whereas patients with benign disease presented with a T helper “Th1-Th17” profile. The latter profile was associated with female gender and a lower mortality rate. Our findings indicate that the most severe cases of COVID-19 are characterized by the presence of overactive immune cells resulting in neutrophil pulmonary infiltration which, in turn, could enhance the inflammatory response and prolong tissue damage. These findings make CXCR2 antagonists, in particular IL-8 antagonists, promising candidates for the treatment of patients with severe COVID-19.

## Author summary

This study was the first to use shotgun metagenomics and in-depth bioanalysis of metagenomics data to understand the role of viral genomics, microorganism metagenomics and host transcriptomics in the severity of COVID-19. The study was performed using nasopharyngeal swabs collected from 104 patients with various severities of COVID-19 at the time of diagnosis. Neither the genetic characteristics of SARS-CoV-2, nor the detection of bacteria, viruses, fungi or parasites were associated with the severity of pulmonary disease. In contrast, host transcriptomic analyses showed that severe COVID-19 pneumonia is associated with overexpression of cytokine transcripts that activate the CXCR2 receptor pathway, whereas patients with benign disease present with a T helper “Th1-Th17” profile. The latter profile was associated with female gender and a lower mortality rate. These findings indicate that the most severe cases of COVID-19 are caused by excess neutrophil infiltration that enhances the inflammatory response and prolongs tissue damage. They suggest that CXCR2 antagonists, in particular IL-8 antagonists, could be promising candidates for the treatment of patients with severe COVID-19.

## Introduction

Severe acute respiratory syndrome coronavirus 2 (SARS-CoV-2) has been identified as the etiological agent of Coronavirus Disease 2019 (COVID-19). The virus emerged in the Chinese city of Wuhan before spreading and becoming pandemic. COVID-19 is characterized by respiratory symptoms of various severities, ranging from mild upper respiratory signs to acute respiratory failure and the acute respiratory distress syndrome (ARDS), with or without fever [1]. Additional, non-respiratory symptoms have been described, including taste and/or olfactory, gastrointestinal, neurological, cardiovascular and/or ocular symptoms [2,3]. The mortality rate of SARS-CoV-2-related ARDS is high. Risk factors for respiratory disease aggravation have been identified, including but not limited to advanced age, male gender, overweight, hypertension, diabetes, respiratory and cardiovascular diseases [4]. Whether genetic characteristics of the virus and/or concomitant bacterial, viral or fungal carriage play a role in the outcome of the disease remains unknown.

The pathophysiological mechanisms of COVID-19 remain largely unknown. Inflammation appears to play a major role in the aggravation of the disease. Anti-inflammatory treatments have been proposed in patients with ARDS, including dexamethasone, which has become part of standard-of-care treatment, and cytokine antagonists. However, inhibiting the inflammatory immune response may also prevent it from clearing the virus, while favoring the onset of coinfections, as already reported for other respiratory viral infections [5].

Metagenomics based on next-generation sequencing is a recently developed high-throughput technology with the ability to sequence the entire genetic content of a clinical sample [6]. This technology can be used to detect known or unknown pathogens [7], reconstruct their full-length genome sequences and/or describe virulence- or resistance-associated motifs [8]. Metagenomics can also be used to characterize host transcriptomic signatures upon the course of any type of infection [9].

The goal of the present study was to use shotgun metagenomics and in-depth bioanalysis of metagenomics data to characterize the genomic, metagenomic and transcriptomic features of patients with various forms of COVID-19 disease, with the aim to identify viral and/or host factors associated with the most severe forms of the disease.

## Results

### Characteristics of the study population and samples

**Fig 1:**
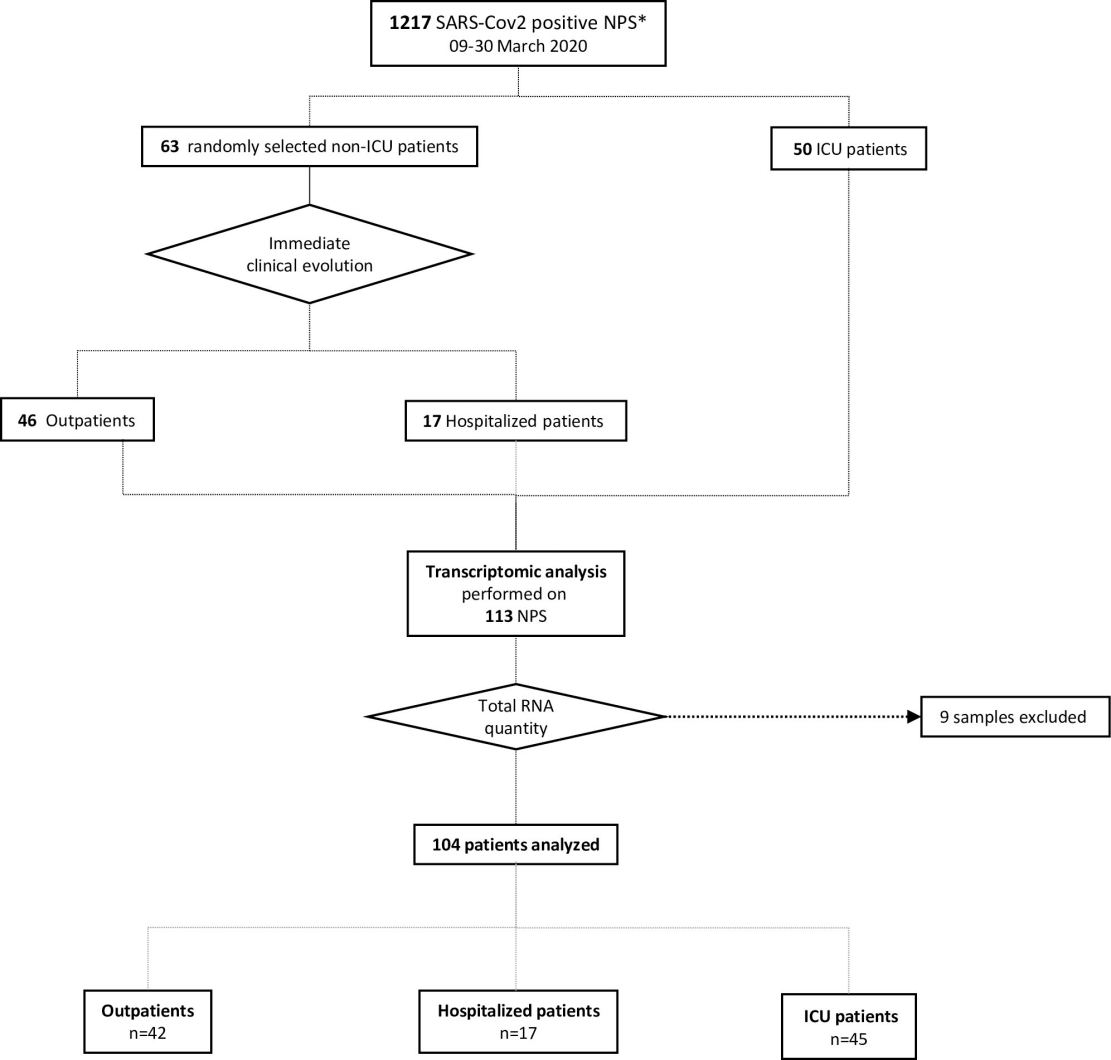
Flow chart of the study.

Overall, 3,254 naso-pharyngeal swabs (NPS) from patients with symptoms compatible with COVID-19 were prospectively tested for the presence of SARS-CoV-2 RNA by reverse transcriptase-quantitative polymerase chain reaction (RT-qPCR) in the Department of Virology of our institution between March 9 and March 30, 2020. Among them, 1,217 (37.4%) were found to be SARS-CoV-2-RNA-positive. Among these 1,217 individuals, the 50 patients requiring hospitalization in the intensive care unit (ICU) with or without mechanical ventilation (“ICU” group, 3.7% of the population) were all included. For comparison, patients not requiring ICU diagnosed during the same period were randomly selected as explained in the Methods section, leading to the inclusion of 46 patients not requiring hospitalization (“Outpatient” group) and 17 patients requiring hospitalization but not intensive care (“Hospitalized” group). The respective number of patients in the latter groups reflected their relative proportion in the exhaustive population. The amount of RNA in the NPS of 9 of the 113 patients from the 3 groups was too small for analysis. Thus, metagenomics analyses were performed in 104 patients (Fig 1).

The characteristics of the 104 patients forming the study population at the time of NPS collection are shown in Table 1. The median (range) ages were 50 (19–87), 61 (31–82) and 68 years (33–90) in Outpatients, Hospitalized patients and ICU patients, respectively (p<0.01). Males were overrepresented in the ICU group (82.2%), compared to 58.8% in Hospitalized patients, and 28.6% in Outpatients (p<0.01). Risk factors for severe disease were significantly more frequent in ICU patients than in Outpatients, including higher body mass index (p = 0.02), diabetes (p<0.01), and hypertension (p<0.01). The median time intervals between the onset of symptoms and the virological diagnosis of SARS-CoV-2 infection were 4 days in Outpatients, 7 days in Hospitalized patients, and 6 days in ICU patients (p<0.01). Three quarters (12/16) of Hospitalized patients required supplemental oxygen, while 46.7% (21/45) of ICU patients required non-invasive ventilation or continuous-positive airway pressure support and 42.3% (19/45) patients required invasive mechanical ventilation. In ICU patients, the median Sepsis-Related Organ Failure Assessment (SOFA) score was 6. Overall, mortality reached 37.8% (17/45) at 15 days post-admission in the ICU group, and 11.8% (2/17) in the Hospitalized patient group, whereas no patients died in the Outpatients group (Table 1).

**Table 1:** Patient characteristics at the time of nasopharyngeal swab sampling.

*Patient characteristics*	*Outpatients (N = 42)*	*Hospitalized patients (N = 17)*	*ICU patients (N = 45)*	P-value*
***Demographic parameters***				
*Age*, *median [range]*, *year (N = 104)*	50 [19–87]	61 [31–82]	68 [33–90]	<0.01
*Female- n/N (%) (N = 104)*	30/42 (71.4)	7/17 (41.2)	8/45 (17.8)	<0.01
***Risk factors***				
*Current smokers- n|N (%) (N = 92)*	4/34 (11.8)	1/14 (7.1)	3/44 (6.8)	0.73
*BMI*, *kg/m*^*2*^ *median [range] (N = 87)*	25.6 [16.9–34.0]	28.1 [19.8–39.0]	28.0 [21.0–43.3]	0.02
*Chronic underlying conditions*, *n|N (%)*				
*Chronic obstructive pulmonary disease (N = 92)*	2/34 (5.8)	1/14 (7.1)	2/44 (4.5)	0.16
*Asthma (N = 92)*	7/34 (20.6)	2/14 (14.3)	1/44 (2.3)	0.03
*Diabetes (N = 92)*	1/34 (2.9)	4/14 (28.6)	14/44 (31.8)	<0.01
*Hypertension (N = 92)*	7/34 (20.6)	8/14 (57.1)	24/44 (54.6)	<0.01
*Cardiac disease (N = 92)*	2/34 (5.9)	2/14 (14.3)	10/44 (22.7)	0.12
*Chronic renal disease (N = 92)*	1/34 (2.9)	0/14 (0)	3/44 (6.8)	0.17
*Cancer (N = 92)*	2/34 (5.9)	0/14 (0)	1/44 (2.3)	0.28
*Immunodeficiency (Transplant*, *HIV) (n = 92)*	0/34 (0)	2/14 (14.3)	4/44 (9.1)	0.33
***COVID treatments*** *n|N (%)*				
*Antibiotics (N = 93)*	11/34 (32.4)	9/15 (60.0)	38/44 (86.4)	<0.01
*Corticosteroids (N = 93)*	1/34 (2.9)	2/15 (13.3)	1/44 (2.3)	0.17
*NSAIDs (N = 93)*	9/34 (26.5)	1/15 (6.7)	5/44 (11.4)	0.11
***Chronic immunosuppressive treatment*** n|N (%) *(N = 93)*	0/34 (0)	2/15 (13.3)	4/44 (9.1)	0.33
***COVID-19 Disease***				
*Interval from symptoms to NSP*, median [range], *days*	4 [0–14]	7 [2–14]	6 [0–19]	<0.01
*Blood neutrophil counts*, *median [range]*, *G/L*		4.9 [2.4–11.0]	5.6 [2.3–16.4]	0.32
*Blood lymphocyte counts*, *median [range]*, *G/L*		1.1 [0.6–3.2]	0.7 [0.2–1.6]	0.01
*Ventilation*, *n|N (%)*				
*Oxygen*	0/42 (0)	12/16 (75.0)	45/45 (100)	<0.01
*Non-invasive ventilation/C-PAP*			21/45 (46.7)	
*Mechanical ventilation*			19/45 (42.3)	
*SOFA score* ≥6, *n|N (%)*			23/43 (53.5)°	
*Death at day-15, n/N (%) (N = 104)*	0/42 (0)	2/17 (11.8)	17/45 (37.8)	<0.01

BMI, body mass index; NSAIDs, non-steroidal anti-inflammatory drugs; C-PAP, continuous positive airway pressure.

* Chi-Square test for categorial data, ANOVA One Way test for quantitative data.

°Blood Pressure was not available for two patients.

### Global results of shotgun metagenomics analyses

Total RNAs were extracted from the 104 NPS and sequenced by means of “shotgun metagenomics”, as already reported. The sequences generated were analyzed with our in-house software MetaMIC [7]. The median (range) depth of sequencing was 34,610,028 sequences per sample (10,868,858–78,673,906), in accordance with the manufacturer’s recommendation (i.e., 5 to 200 million reads). The median (range) phred quality score was 32.5 (30.1–33.9). Sequence bio-analysis generated information on: (i) the genetic nature of SARS-CoV-2 viruses isolated from NPS; (ii) the concomitant presence of bacteria, viruses, fungi, and/or parasites in the swabs; (iii) the nature of host transcriptomic changes induced by SARS-CoV-2 infection.

### SARS-CoV-2 RNA quantification and sequence analysis

**Fig 2:**
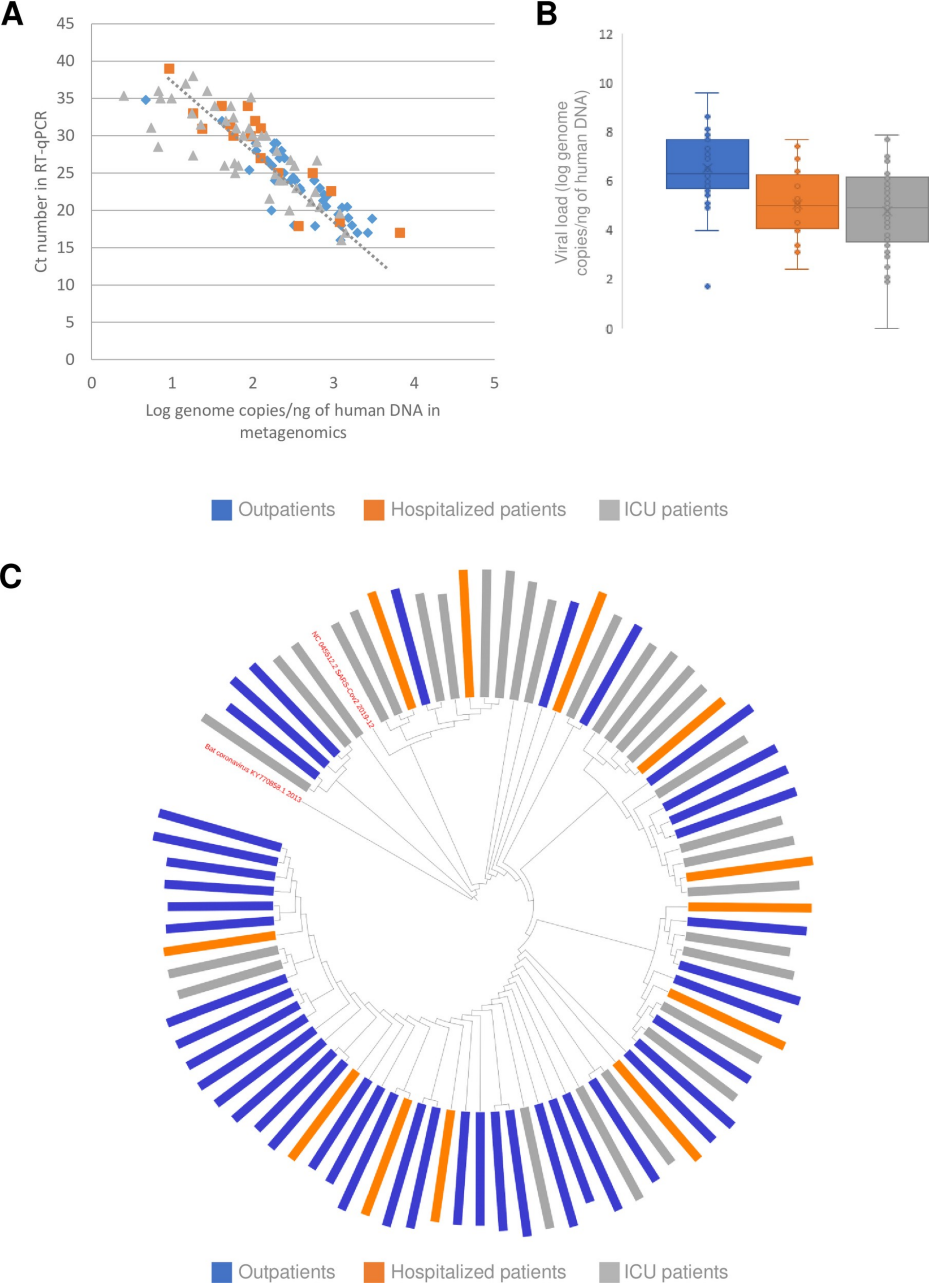
Relationship between SARS-CoV-2 characteristics and the severity of COVID-19 disease. (A) Relationship between SARS-CoV-2 viral loads measured in naso-pharyngeal swabs (NPS) with RT-qPCR or metagenomics. (B) Comparison of SARS-CoV-2 viral loads measured by metagenomics in NPS in the three groups of patients. (C) Phylogenetic analysis of full-length SARS-CoV-2 genome sequences from the three groups (indicated by different colors). *p<0.05; **p<0.01.

As shown in Fig 2A, viral loads measured in Log copies/ng of human DNA in metagenomics correlated with those measured by the Ct number in the same swabs by means of RT-qPCR (r = 0.86, p<0.01). The viral loads were significantly higher in Outpatients than in patients with more severe disease, with no difference between Hospitalized and ICU patients (Fig 2B).

Reconstruction of full-length viral genome sequences was possible in the 74 patients with viral loads >4 Log copies/ng of human DNA, with a median (range) genome length coverage of 99.9% (99.9%-99.9%), and a median (range) sequencing depth of 1,584 full-length sequences (669–141,039). Phylogenetic analysis of full-length SARS-CoV-2 genomes showed no significant clustering according to the severity of the disease (Fig 2C). Bootstrapping values were low (<70%), due to the low genetic diversity of SARS-CoV-2 during the first epidemic wave. No viral signature was associated with the severity of the disease. The reported D614G substitution in the spike protein [10] was present in the 3 groups, significantly more frequent in Outpatients (34/42, 81.0%) than in Hospitalized patients (9/13, 69.2%), and in ICU patients (23/36, 63.9%; p = 0.03). None of the spike substitutions recently described in variants from different geographical origins that emerged during the second epidemic wave (including L18F, Del69/70, Del140, K417N/T, N439K, L452R, Y453F, E484K, N501Y, D681H) were found. Data are available in GenBank (MT470100-MT470179).

### Metagenomics analysis assessing bacterial, viral, fungal, and/or parasitic co-carriage

Metagenomics were used to assess, without *a priori*, the presence of other bacterial, viral, fungal, and/or parasitic microorganisms in the NPS. The proportion of patients carrying potentially pathogenic bacterial agents significantly differed between the Hospitalized group (1/17, 5.9%) and the two other groups: 11/42 (26.2%) in the Outpatients group and 25/45 (55.6%) in the ICU group (p<0.01).

These results were confirmed when considering all pathogens together. Indeed, there was no difference between the Outpatients group (23/42, 54.8%) and the ICU group (30/45, 66.7%; p = 0.26), nor between Outpatients and Hospitalized patients (5/17, 29.4%; p = 0.08), but the difference was statistically significant between ICU and Hospitalized patients (p<0.01). Nevertheless, considering the microorganism species, as shown in Table 2, the 3 groups did not differ for the presence of the different pathogens in NPS.

**Table 2:** Bacterial, viral, fungal and parasite coinfections in nasopharyngeal swabs from the 104 patients, classified according to the severity of COVID-19 and their pathogenic capacity.

	Outpatients (N = 42)	Hospitalized patients (N = 17)	ICU patients (N = 45)
**Potentially pathogenic bacteria (alone or predominant among the oropharyngeal flora or other bacteria)**			
*Haemophilus influenzae*	1	0	1
*Moraxella catarrhalis*	7	0	11
*Staphylococcus aureus*	1	1	6
*Streptococcus pneumoniae*	2	0	7
**Other bacteria not usually detected in the nasal microbiota**			
*Acinetobacter* spp.	0	0	5
*Corynebacterium* spp.	11	5	6
*Lactobacillus crispatus*	0	0	2
*Mycoplasma* spp.	0	0	1
*Pasteurella* spp.	0	0	1
*Pseudomonas* spp.	0	0	2
*Prevotella* spp.	0	0	1
*Proteus mirabilis*	0	0	2
**Pathogenic viruses (alone or associated with bacteria)**			
Human herpes virus 1	1	0	1
Influenza virus B	1	0	1
Mastadenovirus	1	0	1
Respiratory syncitial virus	1	0	0
Rhinovirus	1	1	0
**Non-pathogenic parasites**			
*Entamoeba gingivalis*	0	0	2

### Human transcriptomic analyses

Principal component analysis of global transcriptomic data showed segregation of the patients according to the group they belonged to, suggesting that the expression of certain genes is significantly related to the severity of COVID-19 disease (S1 Fig). Differentially expressed genes, defined by a log2 fold change >2 or <-2 with padj <0.05 (n = 1,910), were used as input to perform unsupervised ranked ontology analysis, in order to identify significant functional enrichments (including up- or downregulation) of genes related to the KEGG pathways. When comparing Outpatients to ICU patients, two pathways were significantly enriched: (i) the cytokine-cytokine receptor interaction pathway, including both CXCR2 and CXCR3 pathways and their main activating chemokines (enrichment score 6.93, FDR 0.0018, KEGG Pathway hsa04060), and (ii) the olfactory transduction pathway (enrichment score 2.69, FDR 0.0081, KEGG Pathway hsa04740).

**Fig 3:**
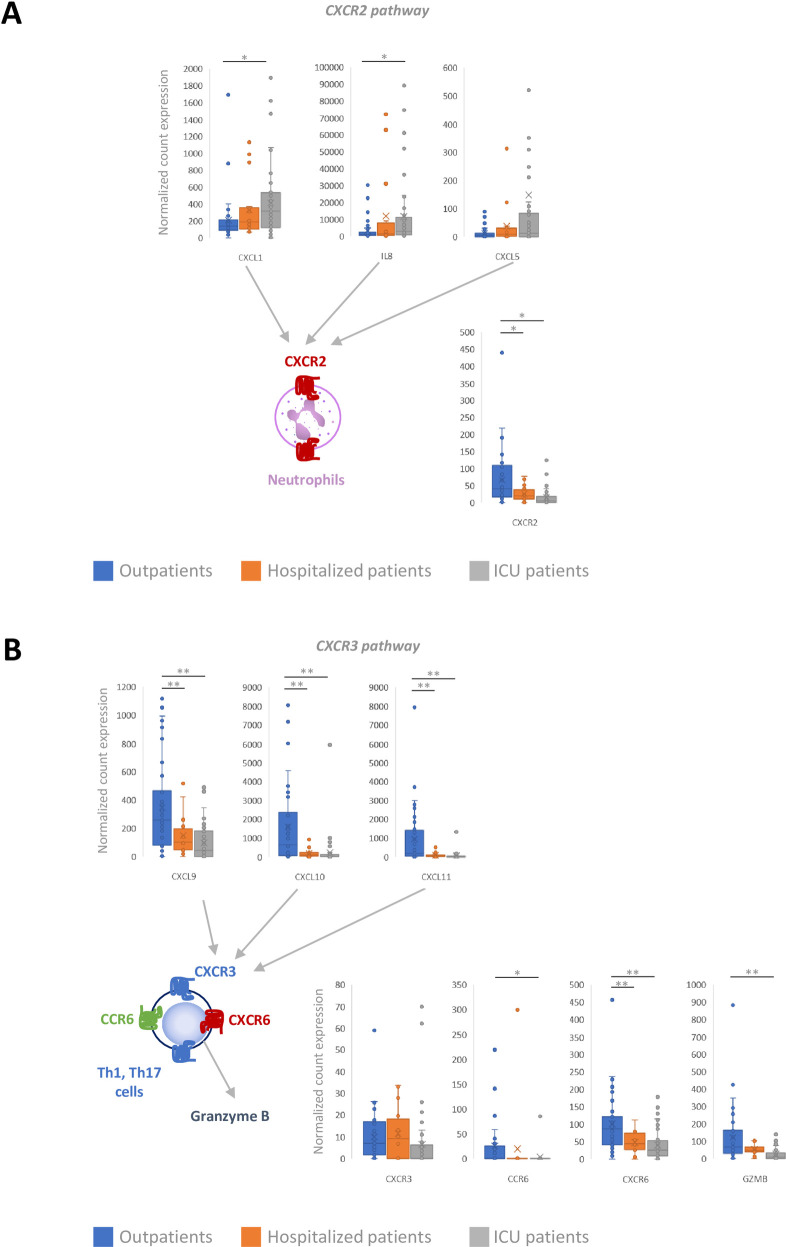
Relationship between naso-pharyngeal swab transcriptomics and the severity of COVID-19 disease. (A) CXCR2 receptor pathway. (B) CXCR3/CCR6 pathway. *p<0.05; **p<0.01.

ICU patients were characterized by a significantly greater expression of pro-inflammatory chemokine transcripts CXCL1 (p = 0.03) and CXCL8 or interleukin 8 (IL-8) (p = 0.04) (Fig 3A). The expression of the CXCL5 gene was also enhanced in ICU patients, but the difference did not reach significance (p = 0.10). Hospitalized patients presented with an intermediate profile (Fig 3A). The expression of the CXCR2 receptor transcript was significantly lower in ICU patients than in Outpatients (p<0.0001) (Fig 3A). Finally, the expression of CCL2, interferon α, β, ε, γ, κ, and λ, and tumor necrosis factor-α genes did not differ between the 3 groups.

Compared to ICU patients, Outpatients presented with a T helper “Th1-Th17” profile, characterized by a significant increase in the expression of CXCL9 (p<0.0001), CXCL10 (p<0.0001) and CXCL11 (p<0.0001) transcripts, that resulted in a significantly enhanced expression of the Granzyme B gene (p<0.0001). Expression of the CXCR6 gene (Th1) was significantly enhanced (p<0.0001), while the increased expression of the CCR6 receptor (Th17) was nearly significant (p = 0.055) (Fig 3B). Hospitalized patients had chemokine profiles closer to ICU patients than to Outpatients for this pathway.

**Fig 4:**
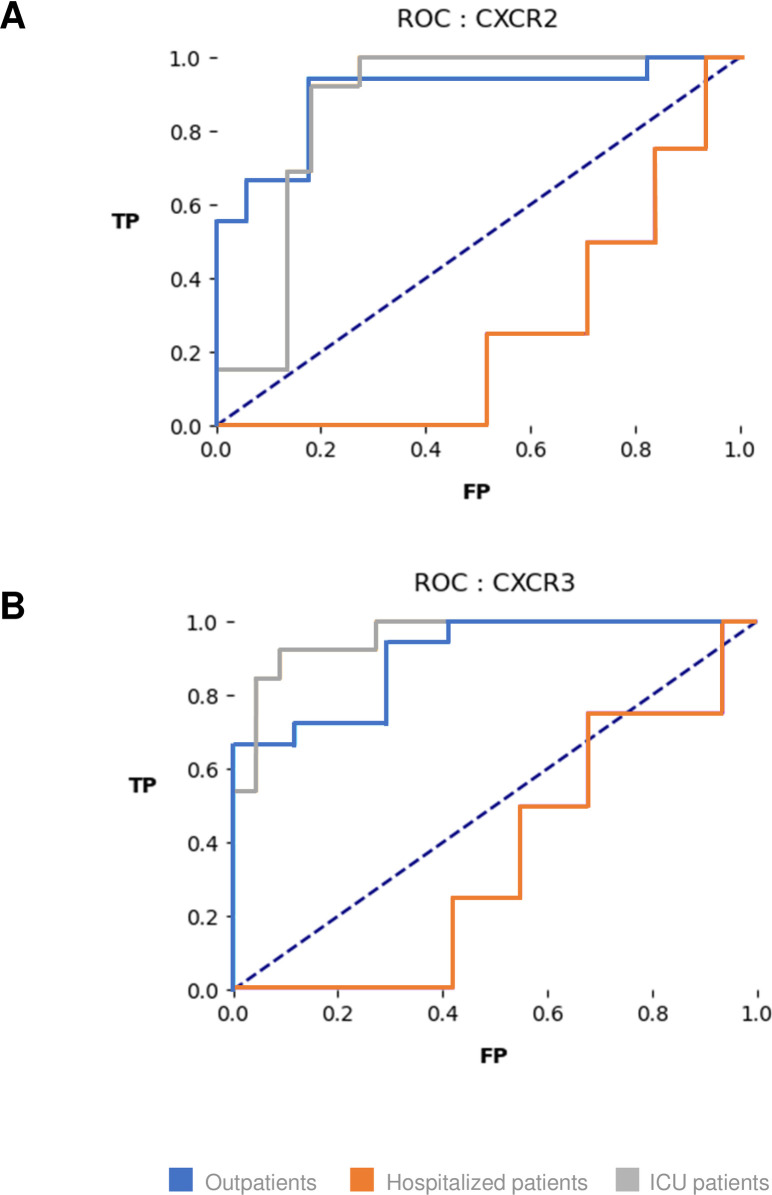
**Receiver operating characteristic (ROC) curves of CXCR2 (A) and CXCR3 (B) panel predictive values on the severity of COVID-19 disease.** The CXCR2 panel included normalized transcript counts for CXCL1, IL8, CXCL5 and CXCR2. The CXCR3 panel included normalized transcript counts for CXCL10, CXCL11, CXCL9, CXCR3, GZMB, CCR6 and CCL20. TP: true positive; FP: false positive.

Receiver operating characteristic (ROC) curves and areas under the curves (AUC) were calculated for each group of patients to assess the predictive value of two panels of genes on the severity of the disease: (i) the CXCR2 panel, including CXCL1, IL-8, CXCL5 and CXCR2; and (ii) the CXCR3 panel, including CXCL10, CXCL11, CXCL9, CXCR3, GZMB, CCR6 and CCL20. As shown in Fig 4A, expression of the CXCR2 panel accurately predicted the severity of the disease (AUC of 0.86 for severity in ICU patients *vs* 0.90 for lack of severity in Outpatients). Conversely, as shown in Fig 4B, expression of the CXCR3 panel accurately predicted the lack of severity of the disease (AUC of 0.91 for non-severity in Outpatients *vs* 0.96 for lack of non-severity in ICU patients). Prediction was poor for Hospitalized patients who had intermediate profiles.

**Fig 5:**
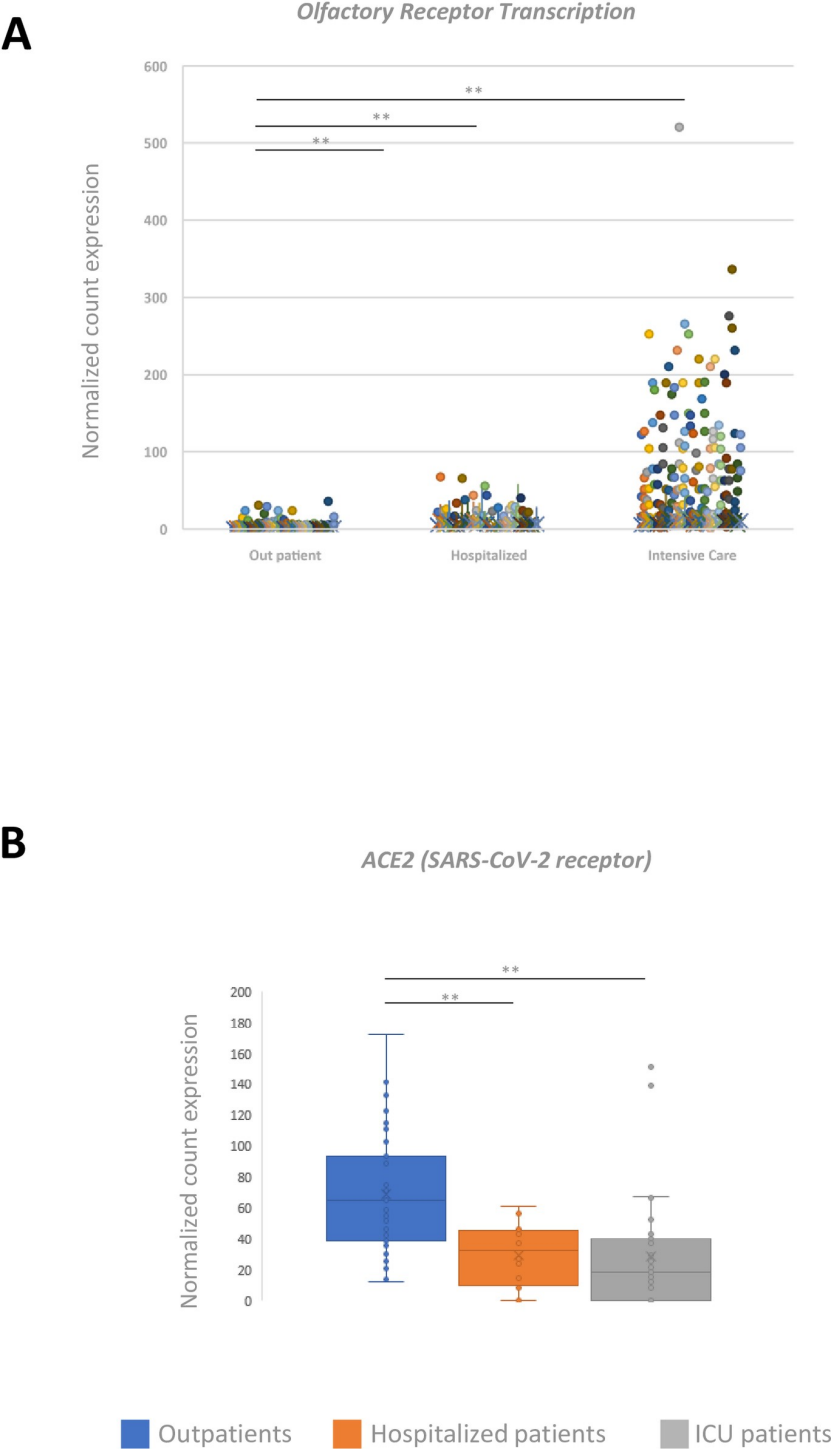
Transcriptomic analyses of olfactory receptors and the ACE2 SARS-CoV-2 receptor. (A) Individual expression of olfactory receptors in the three groups of patients. Each colored circle represents a distinct olfactory receptor differentially transcribed across the three groups (including OR10A7, OR10C1, OR10Q1, OR10V1, OR11A1, OR11H1, OR11L1, OR12D2, OR14K1, OR1I1, OR1J2, OR1L8, OR1M1, OR1Q1, OR2AE1, OR2AT4, OR2B11, OR2F1, OR2G6, OR2T10, OR2V1, OR2W3, OR4D1, OR4K1, OR4M1, OR4Q3, OR51T1, OR52L1, OR52M1, OR52W1, OR56A4, OR6B1, OR6B2, OR6X1, OR8B2, OR8B3 and OR9G1). (B) Expression of the angiotensin-converting enzyme-2 (ACE2) transcript, the SARS-CoV-2 entry receptor, in the three groups of patients. *p<0.05; **p<0.01.

Olfactory receptor gene expression in NPS was significantly reduced in Outpatients, compared to Hospitalized and ICU patients (p<0.0001) (Fig 5A). Finally, angiotensin-converting enzyme 2 (ACE2) transcript expression was significantly greater in Outpatients than in patients with more advanced disease (p<0.0001) (Fig 5B).

### Multivariate analysis and predictors of 30-day mortality

Multivariate analysis showed a positive association between the expression of markers of the CXCR3 pathway (CXCL11, CXCR3, GZMB, CCR6, CCL20), ACE2 and female sex. Two CXCR3 biomarkers (CXCL9 and CXCL10) were negatively associated with age (Table 3).

**Table 3:** Association between clinical factors and biomarker gene expression (n = 93). aOR indicate adjusted odds ratio. Significant associations (p <0.05) are in bold.

			*Age (years)*, *for 1 year increase*	*Female sex (vs Male)*	*BMI ≥30 vs <30 (kg/m*^*2*^*)*	*Hypertension*	*Diabetes*	*Cardiovascular disease*
***CXCR2***	***CXCL1***^***a***^	*Coeff*^*b*^ *[95%CI]*	0.01 [-0.01; 0.02]	**-0.65 [-1.07; -0.23]**	-0.04 [-0.49; -0.40]	-0.16 [-0.59; 0.26]	0.29 [-0.19; 0.77]	-0.67 [-1.23; -0.10]
		*p value*	0.2	**0.003**	0.85	0.45	0.24	0.021
	***IL8***^***a***^	*Coeff*^*b*^ *[95%CI]*	0.02 [-0.01; 0.05]	**-1.25 [-1.99; 0.50]**	0.03 [-0.75; 0.82]	-0.23 [-1.00; 0.54]	0.22 [-0.65; 1.10]	-0.60 [-1.63; 0.43]
		*p value*	0.12	**0.001**	0.93	0.55	0.61	0.25
	***CXCL5***	*aOR*^*b*^ *[95%CI]*	0.99 [0.95; 1.03]	1.07 [0.38; 2.97]	1.17 [0.39; 3.48]	0.94 [0.33; 2.69]	1.69 [0.49; 5.84]	0.45 [0.12; 1.73]
		*p value*	0.6	0.9	0.78	0.91	0.4	0.24
	***CXCR2***	*aOR*^*b*^ *[95%CI]*	1.00 [0.95; 1.05]	**9.17 [1.72; 48.93]**	0.30 [0.08; 1.14]	1.03 [0.30; 3.51]	2.33 [0.54; 10.0]	0.32 [0.06; 1.61]
		*p value*	0.99	**0.009**	0.077	0.97	0.25	0.17
***CXCR3***	***CXCL10***^***a***^	*Coeff*^*b*^* [95%CI]*	**-0.06 [-0.09; -0.03]**	-0.05 [-0.96; 0.86]	-0.84 [-1.78; 0.11]	0.05 [-0.88; 0.98]	-0.42 [-1.54; 0.64]	0.41 [-0.83; 1.65]
		*p value*	**0.0002**	0.91	0.08	0.91	0.41	0.51
	***CXCL11***	*aOR*^*b*^ *[95%CI]*	1.00 [0.95;1.04]	**4.12 [1.11; 15.28]**	0.74 [0.21; 2.66]	1.06 [0.33; 3.44]	1.38 [0.37; 5.11]	0.32 [0.04; 1.46]
		*p value*	0.86	**0.034**	0.64	0.93	0.63	0.14
	***CXCL9***^***a***^	*Coeff*^*b*^ *[95%CI]*	**-0.03 [-0.05; -0.01]**	0.05 [-0.60; 0.72]	-0.35 [-1.04; 0.35]	0.03 [-0.65; 0.72]	-0.43 [-1.20; 0.33]	0.90 [-0.05; 1.85]
		*p value*	**0.007**	0.86	0.32	0.93	0.26	0.064
	***CXCR3***	*aOR*^*b*^ *[95%CI]*	0.98 [0.94; 1.02]	**9.77 [2.95; 32.3]**	4.98 [1.24; 20.00]	0.63 [0.20; 1.95]	4.80 [1.27; 18.10]	1.20 [0.27; 5.29]
		*p value*	0.42	**<0.001**	0.023	0.42	0.02	0.81
	***GZMB***	*aOR*^*b*^ *[95%CI]*	1.01 [0.96; 1.06]	**6.03 [1.36; 26.8]**	1.09 [0.28; 4.21]	0.53 [0.15; 1.87]	3.99 [0.80; 20.00]	0.28 [0.06; 1.43]
		*p value*	0.69	**0.02**	0.9	0.32	0.09	0.13
	***CCR6***	*aOR*^*b*^ *[95%CI]*	0.96 [0.92; 1.00]	**5.53 [1.50; 20.40]**	0.76 [0.20; 2.90]	0.93 [0.20; 4.12]	0.3 [0.03; 2.99]	1.78 [0.24; 13.50]
		*p value*	0.06	**0.01**	0.68	0.93	0.31	0.58
	***CCL20***	*aOR*^*b*^ *[95%CI]*	1.00 [0.95; 1.05]	**5.25 [1.24; 22.10]**	0.73 [0.21; 2.57]	0.97 [0.30; 3.20]	0.96 [0.27; 3.47]	0.54 [0.12; 2.41]
		*p value*	0.92	**0.02**	0.63	0.96	0.95	0.42
***ACE2***		*aOR*^*b*^*[95%CI]*	1.00 [0.95; 1.06]	**22.6 [2.59; 196.40]**	0.78 [0.19; 3.18]	0.48 [0.14; 1.66]	2.47 [0.58; 10.50]	0.28 [0.05; 1.46]
		*p value*	0.94	**0.005**	0.73	0.25	0.22	0.13

a log-transformed

b Multivariate linear regression (Coeff) or logistic regression (aOR) adjusted for all clinical factors

The biomarker predictors of 30-day mortality were analyzed by multivariate analysis. Age, diabetes and obesity were independently associated with death. Thus, multivariate analysis was adjusted for age, sex, diabetes and body mass index. The expressions of CXCL5 (adjusted odds ratio (aOR): 10.50, 95%CI: 1.22–90.55; p = 0.032) and CXCL9 (aOR: 15.48; 95%CI: 1.05–227.12; p = 0.046) were independently associated with 30-day mortality in ICU patients (Table 4). There was also a trend for an association between the expressions of CXCL11 and GZMB and 30-day mortality. No association was found with the other biomarkers (Table 4).

**Table 4:** Multivariate analysis assessing the prognostic value of biomarker gene expression on 30-day mortality in ICU patients (N = 44, 17 deaths).

*Biomarker gene expression (detected vs not)*	aOR*	*95%CI*		p value**
**CXCL5**	10.50	1.22	90.55	**0.032**
**CXCR2**	3.09	0.47	20.25	0.238
**CXCL11**	5.41	0.85	34.38	0.074
**CXCL9**	15.48	1.05	227.12	**0.046**
**GZMB**	5.81	0.86	39.42	0.072
**ACE2**	3.05	0.51	18.24	0.221

aOR indicates adjusted odds ratio.

*Adjusted for age, BMI (≥30kg/m^2^ vs <30kg/m^2^), diabetes and gender.

*Separate multivariate logistic regression for each biomarker

** Wald test

## Discussion

In the present study, we used metagenomics based on next-generation sequencing to characterize the genomic, metagenomic, and transcriptomic features of NPS in patients with various severities of COVID-19 disease, with the goal to identify viral and/or host factors associated with the most severe forms of the infection.

Shotgun metagenomics provides semi-quantification of SARS-CoV-2 viral loads normalized to the amount of cellular materials. Here, the viral loads in the NPS were significantly higher in Outpatients than in patients with more severe pulmonary disease, in keeping with similar observations made during the course of SARS disease at the time of the 2003 epidemic [11]. Based on what is known of the natural dynamics of SARS-CoV-2 viral loads, this finding could suggest that Outpatients are seen at an earlier stage of the infection, whereas severe forms, when they develop, occur later upon the course of the disease. However, Hospitalized and ICU patients were diagnosed only 2–3 days later than Outpatients relative to the onset of symptoms in our study. Together, these results suggest that the severity of SARS-CoV-2 infection is not associated with higher viral loads in NPS.

Shotgun metagenomics allowed us to identify 3 viral co-infections, including influenza virus B, respiratory syncytial virus and rhinovirus, as already described with comparable methods [12]. However, as recently suggested [13], such coinfections are rare. Interestingly, the Hospitalized patient group carried fewer microorganisms, in particular fewer pathogenic bacteria, than the ICU and Outpatients groups in our study. These results are difficult to explain and remain to be confirmed in larger groups of hospitalized patients. Importantly, there was no significant difference in the frequencies of carriage of *Prevotella* sp. genus, found in only one ICU patient, for which a role in the severity of COVID-19 has been suggested [14].

No viral sequence signature was associated with the severity of the disease. The recently described D614G substitution in the spike protein [10] was more frequent in Outpatients than in Hospitalized patients and in ICU patients, suggesting no role in the severity of the disease. Whether this substitution confers a transmissibility advantage remains debated [10]. These results indicate that the genetic characteristics of SARS-CoV-2 have no influence on the severity of COVID-19. Together with the preceding findings, they suggest that differences in severity of COVID-19 are not explained by viral factors. Further studies will be needed to assess whether this holds true with the recently described emergence of new variants in different geographical regions with numerous substitutions in the spike protein.

In contrast, host transcriptomic analyses unequivocally differentiated Outpatients from ICU patients. Transcriptomic patterns from Hospitalized patients appeared to be closer to those from ICU patients. This finding was not surprising, as there appears to be essentially two forms of SARS-CoV-2-induced respiratory disease: a benign form that does not require hospitalization, and a serious pneumonia requiring oxygen supplementation that may ultimately aggravate and require mechanical ventilation. The two pathways that were enriched when comparing Outpatients and ICU patients were the cytokine-cytokine receptor interaction pathway and the olfactory transduction pathway. Severe pneumonia was associated with overexpression of transcripts of cytokines that activate the CXCR2 receptor pathway, including CXCL1, IL-8, and CXCL5, whereas patients with benign disease presented with a T helper “Th1-Th17” profile, characterized by a significant increase in the expression of CXCR3 pathway activator genes, including CXCL9, CXCL10, CXCL11, and Granzyme B. The expression of IL17A, which is produced by Th17 cells and has been suggested to activate CXCL1 [15,16], was not significantly different between the groups.

CXCL1, IL-8, and CXCL5 activate the CXCR2 receptor, the function of which is to recruit neutrophils to the site of inflammation where they play a central role in first-line defences against infection [17]. The involvement of IL-8 in patients with more severe disease suggests that these individuals have reached the amplification phase that prolongs neutrophil recruitment and activation [18]. These findings strengthen the recent hypothesis that the most severe cases of COVID-19 are caused by overactive immune cells producing Neutrophil Extracellular Traps (NETs), excess neutrophil infiltration enhancing the inflammatory response and prolonging tissue damage [19,20]. The expression of the CXCR2 receptor transcript was significantly lower in ICU patients than in Outpatients, possibly because the analysis was global and the CXCR2 receptor is present in many cell types, including epithelial, and endothelial cells, that could be destroyed in large amounts in the context of severe local inflammation. It has also been shown that CXCR2 is downregulated on neutrophils during sepsis, thereby favouring infection [17].

A genome-wide association study identified a gene cluster at locus 3p21.31 as a genetic susceptibility locus in patients with COVID-19 with respiratory failure [21]. Among the 6 genes spanned by the association signal (*SLC6A20*, *LZTFL1*, *CCR9*, *FYCO1*, *CXCR6* and *XCR1*), the CXCR6 gene was significantly under-expressed in ICU patients compared to other groups in our study. No difference was observed for the other genes. CXCR6 expression has been shown to control the pulmonary localization of resident memory T cells, which have been reported to be required for the immune response against different types of respiratory pathogens, such as for instance influenza virus [22]. Together, these findings point to a genetic predisposition to severe immunological forms of COVID-19 infection.

Transcriptomic data, in particular panels of over- or under-expressed genes, can be used to classify patients into different pathophysiology groups. They can also be useful in practice to predict clinical outcomes and make care decisions. In the present study, the expression of panels of genes involved either in the CXCR2 or in the CXCR3 signalling pathways was strongly associated with the severity of COVID-19 disease. Pending validation on another, prospectively recruited cohort of patients with COVID-19, these results suggest that simple gene expression panels in NPS could be used early in the course of the disease to identify patients at risk of severe disease, in order to optimize hospital resources, in particular when an epidemic peak is reached.

Anosmia, and dysgeusia have been reported to be frequent in patients with SARS-CoV-2 infection. They have been more often described in those with a benign form of the disease, while it has been suggested that patients with more advanced disease may under-report this symptom. In our study, olfactory receptor expression was significantly reduced in Outpatients, compared to Hospitalized and ICU patients. This observation confirms the association of anosmia with the least severe forms of infection.

ACE2 has been shown to be the receptor of SARS-CoV-2, mediating its entry into cells through attachment of the spike envelope protein [23]. ACE2 is expressed by olfactory epithelial support cells, but it does not appear to be expressed by olfactory neuronal cells [24]. In our cohort, ACE2 expression in NPS was significantly greater in Outpatients than in patients with more advanced disease. The infection of a larger number of nasal cells in patients with benign disease, that fits with our observation of higher viral loads in these patients, may reflect the early stage of infection. It could explain the greater impact of the infection on olfactory receptor expression and the resulting anosmia in these patients than in those with more severe disease.

Numerous studies have shown that female sex is associated with a better prognosis of COVID-19 disease [25]. It has been suggested to be due to the expression of genes carried by the X chromosome, in particular those coding for the ACE2 receptor [26] and for the CXCR3 pathway of the T-cell response [27]. In our study, most of the biomarkers associated with a good prognosis of infection were associated with female sex, independently of age and cardiometabolic prognosis factors. These results strengthen the hypothesis of a genetic background favouring the severity of the disease. ICU survivors were more likely to express certain markers of the CXCR3 pathway, regardless of the demographic and clinical factors, suggesting the preponderant role of T-cell responses in the protection against severity.

The main limitation of this work is that our studies were performed in NPS and not in lower respiratory specimens from endotracheal aspirate or bronchoscopy with bronchoalveaolar lavage. However, such explorations are unethical because they are unnecessary in outpatients with benign forms of the disease, as well as in hospitalized patients, including those requiring mechanical ventilation, in whom the diagnosis of SARS-CoV-2 infection has been made from NPS. In addition, the American Association for Bronchology and Interventional Pulmonology recently published guidelines indicating that bronchospcopy should be used sparingly in the evaluation and management of patients with suspected or confirmed COVID-19 infections, in order to maximize protection of patients and healthcare workers. Indeed, bronchoscopy is an aerosol-generating procedure that poses substantial risk to patients and staff. It should have an extremely limited role in the diagnosis of COVID-19 and only be considered in intubated patients if NPS are negative for SARS-CoV-2 RNA and other diagnosis is considered that would significantly change clinical management [28]. Therefore, a study comparing genomic, metagenomic, and transcriptomic data in lower respiratory specimens from patients with various COVID-19 severities will never be possible. Fortunately, a recent study comparing the human transcriptomic response in nasal and blood samples from children with acute viral respiratory infection and controls showed that nasal gene expression signatures were as good or better than blood signatures for discriminating between symptomatic, asymptomatic and uninfected children. Therefore, we are confident that our data reflect the systemic host transcriptomic response to infection in the 3 groups of patients studied.

Another limitation of our study is the lack of a SARS-CoV-2-negative control group of patients hospitalized in the ICU during the same period. This group was initially planned in the study design. However, no patients could be included because, like in many other hospitals in the world, our beds, including in the ICU, were entirely occupied by patients infected with SARS-CoV-2 during the study period, which corresponded to the beginning of the first wave of the COVID-19 pandemic in Europe. We then decided not to build a SARS-CoV-2-negative control group during a later period, because both groups would not be comparable due to the seasonality of respiratory infections and the impact of prevention measures put in place in hospitals and the society after the first pandemic wave.

In conclusion, our findings suggest a classical Th1-Th17 T-lymphocyte response in patients with benign COVID-19 disease not requiring hospitalization. In contrast, severe and prolonged inflammation due to neutrophil accumulation plays a major role in severe COVID-19 cases, regardless of the genetic characteristics of the virus or the presence of bacterial, viral, fungal and/or parasitic coinfections. Similar profiles have been reported during SARS-CoV and MERS-CoV infections, but very few human samples could be analysed due to the scarcity of cases during these two epidemics [11]. If the formation of NETs is confirmed, their procoagulant properties could explain the frequency of thrombotic events during the course of COVID-19 disease [29]. These results suggest that immune therapies will be required for the treatment of patients with severe forms of SARS-CoV-2 infection. In this context, CXCR2 antagonists, including several compounds in clinical development [17], could play a beneficial role in the treatment of patients with severe COVID-19. Among them, IL-8 antagonists appear as particularly promising for the treatment of patients with severe COVID-19 in the light of our findings.

## Materials and methods

### Ethics statement

The authors declare that the planning, conduct, and reporting of the study was in line with the Declaration of Helsinki, and French laws for biomedical research. It only involved the use of residual samples that were sent for COVID-19 diagnosis by RT-qPCR and with non-opposition of the patients to their use in an assay. The study was approved by the Henri Mondor Hospital Institutional Review Board (Créteil, France), on approval number 00011558, and all patients provided their written informed consent.

### Patients

All patients seen at the Henri Mondor hospital (Créteil, France) between March 9 and March 30, 2020, with signs compatible with the diagnosis of COVID-19 were tested by RT-qPCR for the presence of SARS-CoV-2 RNA in NPS (RealStar SARS-CoV-2 RT-PCR Kit, Altona Diagnostics, Hamburg, Germany). All patients requiring immediate hospitalization in the ICU were included in the study, while patients in the Hospitalized patient and Outpatient groups were randomly selected among the remaining subjects, who were too many to be all included. For this, the study period was divided into slots of 3 hours, including weekends and nights. Among them, 21 slots of 3 hours were randomly selected, with an overrepresentation (2 to 1) of opening hours *versus* night and weekend hours. All patients sampled during the 21 selected slots were included in the study. Patient characteristics, including gender and age, risk factors for severe COVID-19, treatments and parameters specific for severe COVID-19 cases (in Hospitalized, and ICU patients) were collected.

The characteristics of the patients are shown in Table 1.

### Shotgun metagenomics experimental procedure

The ISO15189-accredited shotgun metagenomics procedure used for this study (MetaMIC) has been previously published [7,30]. Briefly, a pre-extraction step combining mechanical, enzymatic and chemical procedures was performed prior to extraction by means of QIAsymphony DSP DNA Midi Kit on a QiaSymphony Instrument (Qiagen, Hilden, Germany). The extracts were used for library preparation by means of TruSeq Stranded Total RNA kit (Illumina, San Diego, California) and for pair-end sequencing by means of NextSeq 500/550 High Output Kit v2.5 (300 cycles) on a NextSeq500 Instrument (Illumina). A negative control (sterile water) and a positive control (ZymoBIOMICS Microbial Community Standard, Ozyme, Montigny-le-Bretonneux, France) were included in each run and processed through the entire protocol.

### Bioanalysis of metagenomics data

Fastq files were analyzed by our in-house software MetaMIC (v2.2.0). The data were filtered based on quality (phred score) and the sequences were mapped against several custom databases, including human and pathogen sequences derived from the NT database and their annotation. Human sequences were counted, collected in a separate Fastq file and subsequently used for transcriptomic analyses. Microbial sequences were analysed with specific modules. Sequence counts were made for each bacterial, viral, fungal and parasite species and compared to the environmental control. The runs were considered valid if each component of the positive control, that contains a mixture of different microbial species, was detected. The final report included only microbial agents present in significant proportions. For each of them, a specific file containing all annotated sequences was built. This file was also used for SARS-CoV-2 genome analyses.

### Phylogeny

The SARS-CoV-2 annotated sequences from each patient were aligned against the reference sequence (NC_045512.2) with BWA-mem [31]. A consensus full-length genome sequence was obtained for each patient. Multiple alignments were carried out using Muscle [32]; phylogenetic analysis was carried out with IQ-Tree v1.3.11.1 [33], rooted with the KY770858.1 bat virus sequence using GTR+F+I+G4 model and 10,000 bootstraps. The tree was generated by means of iTOL (v4.4.2).

### Transcriptomic analysis

The analysis of transcript expression was performed following previously published guidelines [34]. Briefly, after quality filtration with Sickle v1.33 [35], RNA transcripts were aligned with GRCh37 (release 87) human genome using HISAT2 v2.1.0 [36]. The expression levels of all genes were estimated by means of Stringtie v1.3.5 [37]. Differential expression was calculated using gene count data and DESeq2 version 1.26.0 [38] in R statistical programming environment. Wald test associated with Benjamini and Hochberg procedure were used to test the significance of the differences in transcript expression. Differentially expressed genes with p-values adjusted below 0.01 and log2 fold changes >2 or <-2 were used as input to perform ranked ontology analysis with String [39]. A normalized count of genes was used to identify enriched signalling pathways, taking into account gene ranking according to the extent of over- or under-expression using KEGG Functional pathways [40]. For pathways found to be significantly enriched, the expression of the genes involved was measured from the transcript count data for further comparisons.

To explore the predictive value on the severity of COVID-19 disease of gene expression from the CXCR2 and CXCR3 pathways, normalized count expression of CXCL1, CXCL5, IL8, CXCR2 and CXCL10, CXCL11, CXCL9, CXCR3, GZMB, CCR6, CCL20 respectively was used for analysis with sickit-learn (0.23.2). The dataset was divided in two (2:1), in order to create one dataset to train the model and another one to evaluate it. A linear support-vector classification model was used to fit one classifier per class. ROCs and AUCs were calculated to measure the performance of the two panels.

### Quantification and statistical analysis

Quantitative parametric variables were compared across the 3 groups (Outpatients, Hospitalized patients, and ICU patients) using ANOVA One Way test combined with post-hoc Tukey HSD test for 2-to-2 comparisons when the ANOVA was significant. Qualitative variables were compared with Chi-Square test.

Univariate and multivariate analysis was performed to assess the predictive power of the 12 identified biomarkers on 30-day mortality, independently of the clinical factors. Biomarker gene expression was log-transformed and handled in a continuous way whenever possible (value at 0 corresponding to transcript “not detected”, for CXCL1, IL18, CXCL10, CXCL9), and otherwise transformed into two categories (“detected” *versus* “not detected”). For continuous biomarkers, uni- and multivariate linear regressions were performed to identify clinical factors independently associated with an increased expression of the biomarker gene. For categorical biomarkers, uni- and multivariate logistic regressions were performed. An *a priori* adjustment strategy was chosen, based on known clinical prognosis factors, including age, sex, body mass index, diabetes, hypertension, cardio-vascular disease, and chronic obstructive pulmonary disease. Due to the small number of patients with chronic obstructive pulmonary disease included in the study, adjustment for this variable was not possible. The association between each biomarker and 30-day survival was assessed by uni- and multivariate analysis in the subgroup of ICU patients by means of logistic regression. The same adjustment strategy was used.

All statistical tests were performed using Stata SE (v16.0, College Station, TX, USA). p values <0.05 were considered as significant.

## Supporting information

S1 Fig Principal component analysis of transcriptomic data. Group of severity are indicated in blue (Outpatients), green (Hospitalized patients) and red (ICU patients).
(TIF)
