# Supplementary figures and images for: Viral genomic, metagenomic and human transcriptomic characterization and prediction of the clinical forms of COVID-19
Source: PLoS Pathog. 2021 Mar 29;17(3):e1009416. doi: 10.1371/journal.ppat.1009416 (PMC8032121; doi:10.1371/journal.ppat.1009416)

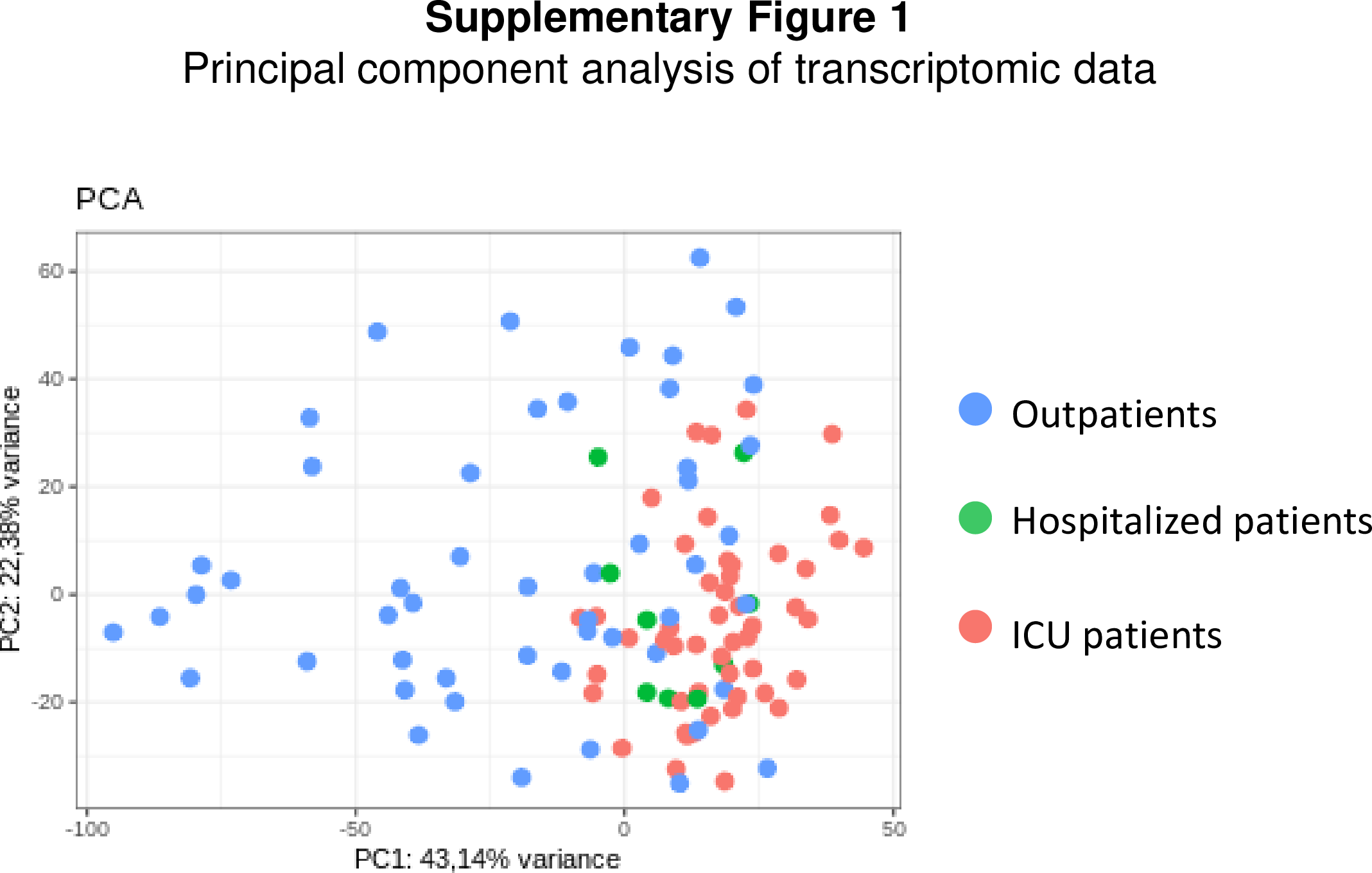

Supplement: S1 Fig — Group of severity are indicated in blue (Outpatients), green (Hospitalized patients) and red (ICU patients). (TIF) [file ppat.1009416.s001.tif]
